# Mental Dynamics in Relation to the Science of Medicine

**Published:** 1854-04-01

**Authors:** M. Lordat, Stanhope Templeman Speer

**Affiliations:** PROFESSOR OF PHYSIOLOGY IN THE UNIVERSITY OF MONTPELLIER; CHELTENHAM


### MENTAL DYNAMICS IN RELATION TO THE SCIENCE OF MEDICINE.

A COURSE OF LECTURES DELIVERED BY M. LORDAT, PROFESSOR OF PHYSIOLOGY IN THE UNIVERSITY OF MONTPELLIER. ARRANGED AND TRANSLATED BY STANHOPE TEMPLEMAN SPEER, M.D., CHELTENHAM.

### LECTURE V.

GENTLEMEN,—I proceed now to an investigation upon which, I confess, I am somewhat loath to enter, but which cannot be passed over. It consists in endeavouring to appreciate the results of the experiments made by zootomists upon living animals, with reference to the functions of the nervous system; results, be it remembered, which they consider as applicable to human physiology.

You may ask why this repugnance on my part? Listen!—These experimenters have, in my opinion, placed themselves beyond the domains of a science which is of ancient date; which possesses its truths, its fixed principles, its links of co-ordination between facts and principles, a familiar acquaintance with the various species of causality, and of the mode in which the different orders of causes are connected with their respective effects. Instead of taking up this science as existing at present, and perfecting it by means of additional facts (allowing always that such facts are capable of connexion with the fundamental subject of the doctrine itself), they have acted precisely as though it had no existence. Not that they have either censured, approved, or examined it; but as though it had never appeared, they have organized a science after their own fashion, without even previously determining its philosophical aspects, and have proceeded to make experiments more or less difficult, with a view to

---

scholar, and his erudition extends through the history of all countries; few men are better acquainted with Eastern literature, and although it is some five and twenty years since we were in the habit of frequently meeting him, it gives us unfeigned satisfaction to learn that he has entirely given up the use of opium, and is in the enjoyment of excellent health."—(p. 109.)

In the November number of Blackwood's Magazine, in an article on "The Narcotics we Indulge in," the author makes a similar statement—namely, that De Quincey completely conquered the habit of opium eating.

ascertain the effects produced upon animals by certain impressions made upon certain organs. From the results obtained by themselves and their copartners in this department, there has been deduced a series of general propositions, which now constitute a species of conventional physiology, the terms and dogmas of which seem to possess nothing in common with the corresponding elements of that science which we here cultivate.

How then can we hope to establish an agreement between two sciences which while in truth not dissimilar, either in a subjective or objective point of view, are nevertheless founded on respective bases of so different a character. How are we to translate the idioms of their science into our own language, when they so carefully avoid a dialect which should be common to us both?

Here, then, in a few words, lies the embarrassment and difficulty which I feel while undertaking the investigation in question. True, it might be suggested that the proper method of procedure would be to trouble ourselves as little about their doctrines as they do about ours. But our respective positions are not identical. The experimental school is composed of those who are either strangers to medical practice, or of practitioners who possess the art of separating the practice of medicine from the science of man, so that the practice itself has nothing in common with the doctrine. We, however, would wish conscientiously to do nothing but what we believe, and to believe nothing but what we *know* to be true.

The necessary connexion between science and practice, will not permit us to regard the experimental school with the same indifference which it affects towards us. Being composed of laborious, talented, and honourable individuals, it is very possible that in the results arrived at, there may be found many things, useful and applicable to the science which we humbly endeavour to expound. It would be wrong, indeed, to expose ourselves to the regret of having neglected a well-established scientific fact, whatever be the source from which it may have sprung.

You must consider the following investigation, then, as an act of resignation on my part.

Do not, however, expect me to follow out, one by one, all the propositions of the doctrine which I combat, as a lawyer impugns all the phrases of his adversary's counsel. A refutation in detail would be too long, and not sufficiently profitable, while it might at the same time degenerate into an acrimonious polemic. I simply wish to draw your attention to the more general notions of our modern vivisector's physiology; less, indeed, with a view of attacking these notions, than of placing them in juxtaposition with the fundamental dogmas of our own science, which, thanks to the contrast, you will be able to comprehend.

A knowledge of the human dynamism has ever been with us an object of profound study. What I have already said with reference to the insenescence of man has afforded us another characteristic, inasmuch as this fact concludes the proofs of the duality of our dynamism, and distinguishes it from that of other living beings.

In comparing the two principles of the human-dynamism, it was necessary to ascertain the rank assigned by philosophy to the vital force.

This retrospective view therefore, of our own doctrines, will constitute a favourable opportunity for drawing a critical parallel between the chief propositions respecting the physiology of the nerves, as taught by the vivisectors of the present day, and those homological propositions of medical physiology which have hitherto been expounded in this amphitheatre.

Thus, the general fact of insenescence, the duality of our dynamism, of which that fact affords a strong proof, and the metaphysical character of the vital principle, are the ideas which I have endeavoured to inculcate; bear them therefore constantly in mind during our ensuing meetings, since they constitute the links, whether tacit or textual, of the subjects which I now propose to consider.

As the science of human nature cannot be made up of *à priori* principles, we would wish it to be based on facts alone. The most ordinary phenomena, of every day occurrence, have taught us that man is composed of an aggregate of the physical order, which we have studied in the dissecting-room, and of a dynamism, which is of the metaphysical order, and which can only be studied in the living, acting man, alternately during sickness and health.

Having considered this first division of our subject, we felt the necessity of entering into the details of the system. We saw the material aggregate to be so constituted, that we do not find in it a single molecule whose presence and mode of existence does not require a power for its maintenance and preservation.

There is, consequently, not merely no apparel or organ in the human body, but not even an atom of whose relations in reference to the dynamism, it is unnecessary to be acquainted with, since the absence of that dynamism is infallibly succeeded by a destruction of the aggregate; i. e., a dispersion of its elements.

But how are we to ascertain these relations, between each part and the dynamism at large, if not by contemplating events as they severally occur?

This contemplation is, in truth, Empiricism, taken in its most general acceptation. It may be carried out in two ways, first, by carefully noticing all that passes, whether accidentally or spontaneously, in the system, and by keeping an accurate account of what there takes place. This is called *observation.* Secondly, by pre-arranging conditions and exciting causes, and then examining consecutive phenomena. This is what has been properly termed *experience.* These two methods, viz., Observation and Experience, are indispensable for the construction and extension of the science of man, and of the natural sciences in general.

The process of observation, which we constantly employ, is after all a slow one. True it is, that with patience and attention, science undoubtedly progresses, but the acquisitions which it makes appear at such long intervals, that we might often be tempted to regard it as stationary.

Experience is more expeditious, and consequently the physical sciences, to which it has been applied, have made immense progress in a short space of time. Unfortunately, its application to our department of science, can be but limited and undecisive. The most conclusive psychological experiments would frequently be attempts made, not merely against the feelings but against the very existence of the subject, and consequently, if man be that subject, the trial must always be barbarous and often criminal.

It has, however, frequently suggested itself to the minds of members of our profession, that certain preconceived and decisive experiments might be made upon animals. But previous to undertaking such experiments they have generally deemed it necessary to reflect beforehand upon the value of the anticipated result; and this reflection has generally been a discouraging one.

To render such experiments profitable, in relation to medical physiology, it would require an amount of analogy between the subject of such experiment and man, as would approach almost to identity. But where are we to find this analogy? Think for a moment and say if it be possible.

In seeking, then, for such analogy, let us examine the whole extent of the inquiry; what is its purport? Is it not to ascertain the mutual relationships, whether active or passive, existing between a given portion of our body, and the dynamism in general? Do we find in some animal an organ anatomically similar to one of our own? we have but discovered half the analogy; we must further inquire whether the brute dynamism, to which this part has reference, bears a like resemblance to our own dynamism. If we cannot discern this double conformity, the results of such experiments may be useful as regards the physiology of that particular animal, but they can serve human physiology, only in proportion to the similarity of the metaphysical terms.

Every living animal is, like man, a combination of diverse materials of the physical order, and of a dynamism of the metaphysical order, investing it with a species of unity, which we term *individuality*. This principle, whether simple or compound, gives laws applicable to its own system. These laws constitute its charts and its codes, which require to be studied in due order, and which it would be rash and imprudent to establish upon mere *à priori* reasoning. Naturalists have pretended to divine the economy of a living being from the outline of its body, but they who have studied the dynamism of such beings, know how faulty these pretended conformities have proved. Nothing is more common than to find species, the internal economy and habits of which are in striking contrast with their external configuration.

From what I have said on previous occasions, more particularly in my lectures of the past year, you cannot fail to perceive how greatly the human dynamism differs from that of the beast.

In the beast, as in man, there exists a vital principle, which is self-acting and spontaneous, void of consciousness, endowed with susceptibility and affectability, plus an aptitude for manifesting these properties in various ways. But this susceptibility and affectability differ greatly, according to the species. The salicor, which proves fatal to man, fattens and improves the condition of the sheep; arsenic, which is poisonous in our case, may be given to a lamb with impunity. With reference, moreover, to the various modes of reaction, they are extremely numerous in man, and are subordinate to the different shades of those affective qualities with which the vital principle is endowed, while in animals they are exceedingly restricted. In some, nothing beyond motion can be elicited; cold-blooded animals appear unsusceptible of inflammation; while, think on the other hand of the prodigious number of diseases produced in man, sooner or later, as the result of external impressions, and you will at once discern the difference between the vital affectability of a human being and that of a brute. For instance, two similar nerves in the same individual, perform *different* functions, and hold varied relations in reference to the dynamism, inasmuch as the vital force has endowed them with different properties; how, then, can nerves of the same name, and more or less similar in the *different* species, be said to possess the same attributes, the same relations, and the same functions, in regard to their respective dynamisms?

The comparative effect of wounds in man and animals has already shown us what a far greater amount of tolerance (ευφορια) exists in the latter, since what is denominated by surgeons, traumatism (βλάϐη of Galen), is almost unknown in animals, as a consequence of even the severest operations.

We say nought of the degree of vital tenacity, which is extremely variable in different species, without apparent reason. Few animals are cosmopolitan, man alone appearing to possess this privilege. On the other hand, he not unfrequently dies of comparatively limited injuries, while in the tardigrade, called the *Unau*, it is extremely difficult to extinguish life.

Does there exist in animals that relationship between certain organs which we designate sympathy, and which is of so much importance in medical practice? the question is still *sub judice*.

The vital force in man is endowed with a certain degree of instinct or aptitude for performing directly and involuntarily, various automatic acts.

You are aware that respiration, suction, deglutition, the expulsion of excrement, &c., are performed before reason or volition have assumed the initiative. We are especially convinced of the automatic character of these acts, inasmuch as they have been observed in living human monsters, void of either brain or spinal cord—monsters that have been called Amyelencephales, and in whom it would be impossible to conceive the existence of an intellectual principle.

A comparatively recent occurrence confirms what I have just said. You will find it mentioned in M. Longet's *Anatomie et Physiologie du Système Nerveux*. "A female, with contracted pelvis, became pregnant; after various ineffectual attempts at delivery with the forceps, it was determined to perform craniotomy. Doctor Beyer (who narrates the case) immediately did so, extracted the two parietal bones, emptied the cranium, and removed the child, which was then wrapped in a napkin and thrown into a corner. While the medical attendant was engaged in removing the placenta, he heard a species of murmur, emanating from the spot in which the child had been deposited. In a few minutes a distinct cry was heard. The napkin was opened, and to the astonishment of all present, this acephalous fœtus was breathing, and throwing about its arms and legs; it uttered several cries, and gave sundry indications of life, during the space of several minutes." Bear in mind that the skull was empty, and that consequently the rachidian bulb had no longer existence. This did not, however, prevent the child from breathing and crying.

We have here more than enough evidence to prove the nature of instinct, and its existence as independent of the intellectual principle, but dependent on the vital force. The amount of human instinct, however, is but trifling in comparison to that with which the majority of animals are endowed at the moment of birth. What acts, what functions of relation, do they not perform without need of trial or apprenticeship? This exaltation of an instinctive faculty, of whose existence we are fully convinced, renders it impossible for us to decide whether there be ought but one grand instinct engaged in the exercise of the functions of relation, as occurring in the brute. Spite of our doubts, we nevertheless do not question the existence in animals of a true sensibility, a susceptibility accompanied by self-consciousness. But what is this animal sensibility as compared with that of man? In the former it bears reference to the interests of the vital system alone. A sensation is always either pleasant or painful, that is, favourable or unfavourable to the aggregate; and we know not whether that which does not bear reference to this species of interest, can truly be denominated a sensation. This sensation serves as a guide to the instinct, and nothing further. We have no grounds for believing that it can impart instruction, or produce a combination of ideas from which a thought might suggest itself. It serves to entertain a species of animal memory (if I may so speak), which the instinct recals at need, whenever the varying conditions of the vital principle render such an act necessary.

Compare, then, this sensibility with that which we possess. The latter is doubtless roused by impressions, whether from without or from within; but as our instinct is so imperfect, it is needful that the intellect should also appreciate the sensation, in order to know how it is to be forthwith responded to. Thus it is, that sensations are in reality wants, which oblige us at a very early period to think, and hence serve as instructors. What a difference have we here, between this species of sensibility, and that of the lower animal at the moment of birth.

Buffon has addressed to us a serious reproach for not attempting to compare accurately the human dynamism with that of the brute. "It is not to be wondered at," says he, "that man, who knows so little of himself, who confounds often his sensations and ideas, and who distinguishes so imperfectly the emanations of the soul from those of the brain, should compare himself to the brute, and admit, between it and himself, nought but a shade of difference, depending upon a trifling excess or deficiency in the perfection of his corporeal organs. It is not surprising that he should assert their powers of reasoning, of mutual comprehension and of self-determination, and attribute to them in addition, not merely the very qualifications which he himself possesses, but even those in which he is deficient. But let man examine and analyze himself thoroughly, and he soon will recognise the nobility of his nature, comprehend fully the existence of his own mind, cease to debase himself, and perceive at a glance the infinite distance which the Supreme Being has interposed between himself and the brute."

This exhortation on the part of the great naturalist, is all but lost upon the school of the Organicists. Cabanis asserts that every sensation produces either pleasure or pain—true, for the brute ; to whom sensation is as nothing unless it be immediately conducive or injurious to the maintenance of the aggregate. But, as regards ourselves, how many impressions are neither painful nor pleasurable, but on the contrary, a source of absolute indifference ; while, so far as our moral interests are concerned, their appreciation most undoubtedly depends neither upon sensation nor upon instinct, but solely upon reason.

When our sensations are pleasurable, have they aught in common with those of the animal ? The animal enjoys them to the utmost, until they have become exhausted. We carefully avoid doing this, and have invented a thousand refinements, if not to augment (for our sensibility has also its limits), at least to prolong and surround them with a species of intellectual value which increases their importance to an almost indefinable extent. We possess an æsthetic for each sense. There is not one, on whose behalf volumes have not been written, in order to multiply its modes of susceptibility, to anticipate pleasure by prevision and preliminary, to increase attention at the moment of sensation, and to prolong the agreeable rememoration of the same. Is there, then, any similarity between these methods of procuring pleasure and the sensation of the brute ? The difference truly is so great, that I scarcely venture to apply the term *sensation* as expressive of the conscious susceptibility to external impressions of both man and animals.

Here the comparison between the two terminates: the principle of thought, which is all in all with the former, gives no evidence of existence in the latter. I can only feel certain that a living being thinks, when he can communicate to me his thoughts by a conventional language, whether phonic or aphonic, as I mentioned to you during the past year. The education, so to speak, of the brute, is not identical with ours, since with us, education is a process of instruction, taking place in the intellectual principle ; while in the brute, education is merely a mode of forming and fashioning the instinctive propensities ; a mode, indeed, which constitutes a species of type, as transmissible by generation as an ordinary morbid process. Finally, do we find in the life of the animal aught that calls to remembrance the coincidence of a principle which, after acquiring its utmost development, becomes aged and degenerate, with another principle possessing the power of self-preservation, and capable of becoming an intelligent witness of the destruction of its own tenement and of the senile extinction of its *biotic* companion ? Does the brute enjoy a mental insenescence, contemporaneous with the senescence of its vital principle ?

No ! a principle of intelligence, such as I see and study in man, is not that which animates the brute ; and this constitutes, in my opinion, the most striking disparity between the human and the bestial dynamism.

There is, moreover, in connexion with man, a subject which appears to be of great scientific interest. I allude to the laws which regulate the alliance existing between the vital and intellectual principle during the whole course of life ; an alliance in which we find, at one time an increased, at another a diminished, amount of co-operation ; while occasionally we discern a natural suspension, and even an actual aberration, in their association. This important item of human physiology, constituting as it does, the basis of all theories respecting sleep, somnambulism, delirium, intoxication, morosophy, and mental alienation, belongs to the science of man alone. You will find nothing in zoological physiology, at all analogous to the facts of which I speak.

It might perchance, however, be objected, that the phenomena designated as sleep and asphyxia in animals, are similar to what take place in man; but I trust ere long to prove that it is not so. In ourselves for instance, the transition from a sleeping to a waking state convinces us that the co-operation of the two principles has been temporarily suspended, and that at the moment of transition the co-operation is renewed. A similar occurence takes place upon recovering from the attack of some ecstatic disease, from magnetic sleep, or from asphyxia. In every instance of the kind there is a moment of astonishment. "*Where am I?*" either escapes the lips or flashes across the mind. In the feigned fainting fits occurring during the representations of the drama, the individual never fails to express this surprise, which is in truth the inevitable result of an interval, existing between the previous estrangement of the two principles and their complete reunion. A sudden waking out of sleep is always accompanied in man by indecision. I do not believe, however, that this is noticed in animals.

The recovery from a state of asphyxia is never accompanied in man by phenomena similar to what we notice in the recovery of a bird under similar circumstances; as in the experiment of Humboldt, made with a view of testing the effects of galvanism. After having had recourse to the necessary means for producing asphyxia, "I waited," says he, "for the moment at which the subject of experiment, a common linnet, was about to expire. Already its eyes were closed, and it was stretched on its back, while mechanical irritation with the point of a pin in the vicinity of the anus, produced no effect. I hastened to place a piece of zinc in its beak, and another of silver in the anus, and immediately afterwards a communication was established between the metals through the medium of an iron wire. To my astonishment, at the moment of contact, the bird opened its eyes, raised itself upon its feet, and fluttered its wings; it then breathed for six or seven minutes, and expired tranquilly." There was nothing in the case of the bird at all corresponding to the "Where am I!" so invariable in man under analogous circumstances.

After such evidence of discrepancy existing between man and animals, what expectations can we found upon the experiments of vivisectors; especially when the theory of the animal functions is the question at issue? The similarity actually existing between the two terms of comparison, viz., the animal and the human dynamism, does it constitute anything like analogy? The terms, it is true, are both of the *metaphysical order*, but how far removed from one another!

Such are the reasons why we place but little confidence in the results of vivisection. It may, however, be said—" Suppose that man be an animal, to which an *intellectual* principle has been superadded, remove this principle, which we admit will produce an enormous difference, still the object of this addition is an animal, like others, and you cannot avoid recognising the analogy." But to this I would reply, as I have previously done, that the *vital* element in man differs from that of animals, in its laws, instincts, and susceptibilities. Remember, moreover, that the human vital principle was created the coadjutor of the intellect; and can we imagine that such an auxiliary should be a vital principle similar to that for which in the brute instinct alone suffices to be the companion?

Lastly, if animals really possessed such an analogy to the human species as that their respective physiologies were identical, if their dynamism resembled ours, if they possessed the same susceptibility that we possess,—an intelligent principle, similar to that of the child, or even of the savage, who, according to the assertion of some naturalists, possesses less mental capacity than the elephant or the monkey,—should we have dared to resolve the problem by means of the scalpel? If their sensibility were identical with that which we possess; if pain, inflicted by the knife, produced in them, trembling, terror, fear of danger, and visions of approaching death, should we I ask, ever have had recourse to a sanguinary experiment? We should, indeed, have feared to become fratricides, and rather have wished the animal to be treated as we would wish the negro to be treated. We have, therefore, but little sympathy with vivisection. If authorised by analogy, it is both criminal and ferocious; and if justified by a difference of nature, it remains objectless and unworthy of confidence.

Knowing then, the reasons which actuate me in the rejection of vivisection as a means of illustrating the human dynamism, it becomes necessary to inquire into the motives of those experimenters who devote themselves with so much zeal to this species of investigation.

And first let me remind you that, with a view of demonstrating the results we may anticipate from experiments made upon animals, I began by discussing our intellectual requirements, our tendencies, our philosophy, and the bias of our mind in the study of medical physiology.

To complete this portion of our subject, let us endeavour to study the vivisectors in the same light; let us see what is their object, what are their preliminary studies, their inclinations, their philosophy, and their mode of applying it.

While then, we, on the one hand, have openly avowed the motives which actuate us, the vivisectors have been much less explicit; and we must, therefore, endeavour to unravel their ultimate designs.

I. I have already stated that the essential object of our studies is the knowledge and legitimate practice of medicine. The importance, therefore, of this profession, authorizes an inquiry into whatever pertains to it; but repudiates all serious efforts of the mind directed to that which is foreign to its interests: being, in truth, a science, of which it may be reasonably said that no servant can serve two masters.

The vivisectors have, however, adopted an additional vocation; they are essentially naturalists. In examining the acts of an animal, they have had the curiosity to inquire into the mechanism of its movements. This is far from what would have been our design, had we become vivisectors; our objects would rather have been anthropological. The difference of the impulsion must then necessarily show itself in all the results accruing from the practice in question.

Our primary course of study, then, has been that prescribed by our model, Hippocrates, viz., an inquiry into human nature. It is from constant attention to this subject, during a period of more than two thousand years, that there has arisen that Hippocratic vitalism which practitioners of medicine tacitly cultivate; and which our own faculty endeavours to preserve, perfect and propagate, with the utmost zeal. For this doctrine the vivisectors have no sympathy: not merely do they ignore its essential doctrine, but they conceive it, moreover, to be other than it actually is.

They nevertheless assert that their efforts are directed to the advancement of medical science; and, in truth, they strenuously endeavour to introduce their results within its domain. This is at least a mode of giving importance to their investigations, of which it remains for us to appreciate the intrinsic value.

Let us not, however, break off all intercourse, merely on account of their distaste for vitalism; such repugnance is rather a reason for hearing them; inasmuch as certain of their results, which actually fortify our own doctrinal views, cannot be suspected of favouritism, always indeed to be dreaded in the recitals of a friend.

You are aware that our mode of procedure, in the research after natural truths, is that propounded by Bacon in his "Novum Organum." It is that which has always served as a rule in the construction of the Hippocratic doctrine. We start with a number of anthropological facts; and after having classed them according to their mutual resemblances, we ascribe to each class a cause, of which the name expresses but the effects. These names stand for the experimental causes. When the groups are multiple, we compare the different experimental causes, in order to combine those which a more attentive examination has proved to be identical; we segregate those which possess peculiar characteristics, and which it would be impossible to confound, until new facts shall have taught us that experimental causes, different in aspect, may nevertheless be referred to one common cause already known. It is thus that modern chemists proceed: they fear not to multiply experimental causes, apparently distinct, and to give them specific names . . . . comparing them only when the number and variety of ascertained facts shall have definitely settled their proper rank and position. These rules have been established with a view of banishing from science all hypotheses and suppositions, inasmuch as they encumber it to no purpose.

The terms, vital force, instinct, susceptibility, irritability, automatism, sensibility, innate principle, volition, human dynamism, &c., have been employed, merely to distinguish the various groups of anthropological phenomena, and to admit of their respective causes being definitely established under certain heads.

The experimenters of whom I now speak, disdain such philosophy; rejecting all experimental causes, ascertained by abstraction alone, they require something more consistent and more corporeal. In default of well-marked physical causes, they willingly content themselves with a very concrete hypothesis, imitating somewhat those savages who, in case of famine, fill their stomachs with earth, which, though in itself useless, serves nevertheless to amuse the organ, at least for a time.

Thus, they have assumed that the nerves and ganglia animate the whole body, "and regulate the functions of the entire economy." But the nerves, as they come under our inspection, do not appear particularly adapted to such a purpose. Again, "in *order to explain in man and animals the phenomena of physical existence* (*this, be it remembered, is their peculiar mode of expressing themselves*) *the majority of authors admit the presence of an imponderable agent, designated by the various titles of, nervous fluid or agency, nervous power, acting principle of the nerves.*" They are at a loss to know whether it be identical with the electric fluid, or whether it be a fluid *sui generis;* but they cannot do without some imponderable of a physical order.

M. Longet, following the example of M. Muller, has discussed this complex question at considerable length; he fails to resolve it, but lest the mind should lose all hope of discovering a fluid similar to others, belonging to the domain of physics, he thus sums up his chapter:—

1st. "There is no direct proof in favour of the hypothesis, that currents of electricity pass along the nerves.

2nd. "Electricity and the nervous power are not identical.

3rd. "In the present state of scientific inquiry, it would be rash to assert that they are totally different, and possess no analogy with one another."

But I would venture to ask, If there be any great rashness in asserting that the force which produces zoonomic life and the fusiform phenomenon previously described at length, has no radical analogy with electricity?

This partiality for hypothesis, against which Bacon has directly protested, finds support in the example of Descartes, and in that remarkable maxim of his "Natural Philosophy," which appears to me to be fraught with dangerous results, and which, if taken literally, would convert science into romance—"I should feel," says he, "that I had done enough, if the causes which I have explained should appear to be of such a nature as to produce effects, bearing even a resemblance to those, which we witness around us in the world at large; without inquiring whether it be by these, or by other causes, that such effects are produced. I conceive, moreover, that it is as useful to possess a knowledge of these supposititious causes, as of the actual ones." Hereupon he quotes a passage from Aristotle, which appears to support his views. But I again ask, Can we say that science consists in seeking indifferently for what is true, or for what is merely probable? Happily it is not so before a court of assizes. A crime has been committed, an individual has been accused; the jury is asked whether they feel confident that the said individual is guilty. The answer is not, that his guilt appears certain or probable, it must either be the expression of conscious conviction or silence; and why should it not thus be with regard to science? If not sure of the cause of such and such an effect, you are not obliged to adopt one that is simply probable. Withhold your judgment, and seek more ample information.

III. The distinction between the two classes of causes: viz., those of the *physical* and those of the *metaphysical order*, in the sense understood by Bacon, is rigorously observed among us, as I have already had occasion to observe. It has been stated, that the *metaphysical order* is accepted in this sense, as a category of the natural sciences, having no connexion with the theological sciences. This observation is necessary, inasmuch as, during the Cartesian epoch, metaphysics and theology were associated; while certain modern philosophers of the Epicurean sect take advantage of this past association to deny metaphysics, and to treat them as a mere superstition.

True it is, that in Paris metaphysics have been limited to pyschology, and pyschology in turn connected with religion. The allegorical tableaux of the latter part of the seventeenth century represent the science of metaphysics as identified with theology. I have already brought this under your notice in the frontispiece of Diderot and d'Alembert's Encyclopedia; and I have now before me an engraving of Bernard Picart, published in 1707, the title of which is, *Truth as sought after by Philosophers.* Truth is seen in the distance, while Philosophy is pointing it out to her favourite, Descartes, and to the philosophers of antiquity. The allegorical female figure, representing Philosophy, is adorned with certain attributes, symbolical of its four sub-divisions. She is crowned with stars, in order to designate physics; she holds in her right hand a sceptre, emblem of morals; in the other she holds a serpent, with its tail in its mouth, a symbol of eternity, intending, moreover, to represent metaphysics; while she places one foot upon a touch-stone, to indicate logic, the object of which is to discern truth from falsehood.

This association of metaphysics with the idea of eternity, of the Almighty, and of his relations with man, is an amalgamation whose intent in those days was morally excellent; the connexion is now, however, maintained with perfidious intent.

Let us then revert to Bacon's division, and distinguish physical from metaphysical causes, in that the former first produce their effects without fail or variation, under the same evident conditions; secondly, that they act without relaxation or exaltation of intensity; thirdly, that they are as durable as the bodies from whence such causes spring; fourthly, that they produce phenomena, each of which is isolated and independent both of the past and of the future: while the latter order of causes, viz., the metaphysical, are, first, adventitious as regards their locality, variable in their effects, and consequently prone to contingency; secondly, they are subject to remissions and exacerbations, without extrinsic determination; thirdly, that they are forced to execute a series of successive phenomena, and are condemned to inaction when these have been achieved, in spite of the aptitude of locality; fourthly, that they give rise to phenomena which are connected, like the links of a chain, not of necessity or in a manner physically indissoluble, but by conventional agreement, with that temporary period, each moment of which has relation to its beginning and end. In a word, let us recollect that the causes of the physical order act *ratione entis*, and those of the metaphysical order, *ratione moris*.

It would appear that the vivisectors generally, have not the slightest notion of this distinction. I have already said that there are some among them who conceive these to be *words without ideas ;* but I would ask my auditors if the above distinction be nought but a tissue of words, void of meaning?

Sustaining the character of staunch organicists, they persist in seeing, in the parts upon which they operate, nothing but what comes under the cognizance of their senses, plus the nervous fluid which they have invented. They, nevertheless, occasionally make use of words expressing agencies which they well know not to belong to the physical order. Thus, M. Muller appears to recognise a *principle of life, a mind.* And I have already stated that he expresses his opinion of the unity of the dynamism, both in man and animals, so that he adopts a reversed Stahlianism—one single acting cause, one vital force, in which the principles of the intelligence is but an additional faculty. M. Longet, moreover, speaks of *life* and of the *intellect,* which he does not undertake to explain by physics or by chemistry. The theory of an alleged nervous fluid, is termed mechanical; this expression, so rarely employed in reference to the doctrine of the imponderables, and of which the true signification in physics is well known, appears to be doubly incongruous when physiology is the subject under consideration.

The vivisectors imagine that the nervous fluid is produced in the brain and spinal cord, without considering that the vital force is anterior to the formation of the nervous system. The movement of this fluid constitutes the chief subject of their theory, and its authors carefully endeavour to unite in one, both the idea of the phenomenon and that of its alleged cause. Let us here cursorily examine the terms which they employ, in order to express these facts, with the view of supporting their hypothesis.

1st. They assert that there are three species of nerve fibres, of which the first are denominated sensory; the second, motor; the third, excito motor. Each nerve fibre irrevocably possesses certain properties in virtue of its primitive constitution. They start from the periphery, and unite in the cerebro-spinal centre.

In man, an impression, made upon any point of the living system, is felt either by the vital force alone, or by the two principles simultaneously. The former of these phenomena constitutes *susception* or *vital sensation;* the latter is sensation, properly so called. These terms are but the expression of certain facts. The experimentalists call them *centripetal acts.*

When a susception or a sensation takes place in the dynamism, the event is appreciated either for good or evil. An impression is received, which is either favourable or not, and there results as a consequence, an act which expresses the nature of this appreciation. Physicians say that there exists in some part of the dynamism, an irritation, an affection, &c., and the appreciation of the same is expressed by terms, which differ according to its mode of manifestation—*fainting, convulsion, inflammation, instinctive motion, voluntary motion,* &c; such are the facts. The experimental school designates this appreciation as a conflict between the motor and sensory nerves at their central point of union. With regard to the expressions made use of, in reference to the appreciation of which the motor nerves are capable, they are comprised in the term *centrifugal* acts.

Thus, at every page of their writings do we find a translation into the language of physics, of medical terms, derived originally from that of metaphysics. Nevertheless, there are certain facts which have refused thus to accommodate themselves. We shall have, ere long, occasion to notice these more particularly; it will, however, suffice to show, that by their own acknowledgment, the vital principle is not of the physical order, and that it acts *ratione moris,* and not *ratione entis;* witness that *solidarity* of organs recognised by M. Longet, a characteristic indeed which is unknown in the domain of physics.

V. This conviction, or at least this attachment of the vivisectors to their own peculiar tenets, together with the liberty which their philosophy allows of creating hypotheses indefinitely, of dispensing with the necessity of seeking for what *really* is, and of contenting themselves with putting forth that which *might* be, is a frame of mind which appears to be very suspicious, and against which we should be on our guard. It gives me, moreover, a certain distrust of their anatomical discoveries. As these are made only through the medium of the microscope, and as this method of investigation may give rise to many illusions, how can I avoid receiving them with some hesitation, when I find them to be at variance with recognised dogmas?

To establish their doctrine, it should be shown that each nervous filament is independent of its neighbour, from the cerebro-spinal centre to its periphery. . . . General anatomy, however, shows us the anastomoses of the nerve fibres; appearances, therefore, are in favour of the latter.

Again, their doctrine requires that the nerves of the ganglionic system should accompany the blood vessels to their finest ramifications, but the demonstration of this fact is impossible.

In order that the phenomena of innervation should appear to be in accordance with the varied conditions presented by the anatomy of the nerves, they have supposed that the grey matter might explain what has been wanting to their theory; and, consequently, efforts have been made to make of this tissue a system similar to that of the nerves themselves . . . . . an attempt by no means easy, if faithful to the first precept of anatomy, viz., demonstration.

As for myself, these portentous anticipations appear to have little reference to the fundamental principles of science. Be they as well founded and susceptible of the most unmistakable demonstration as possible, the probability of the doctrine of the vivisectors would not be enhanced, nor that of ours diminished. The different parts of the material system are but the machinery of which the power and the modes of action are known by certain facts. We shall learn with pleasure every detail concerning the aptitude and characteristics of this machinery, but such knowledge will change nought of what is essential to our scientific edifice. It is not so, however, with their hypothesis. It can only possess a semblance of reality, upon the supposition that this new anatomy should prove to be true. If they succeed in their anatomical enterprise, the hypothesis may still be maintained, but simply as a supposition.

